# Impact of Aflatoxins on the Digestive, Immune, and Nervous Systems: The Role of Microbiota and Probiotics in Toxicity Protection

**DOI:** 10.3390/ijms26178258

**Published:** 2025-08-26

**Authors:** Katarzyna Chałaśkiewicz, Katarzyna Kępka-Borkowska, Rafał Radosław Starzyński, Magdalena Ogłuszka, Mateusz Borkowski, Ewa Poławska, Adam Lepczyński, Elżbieta Lichwiarska, Sharmin Sultana, Garima Kalra, Nihal Purohit, Chandra Shekhar Pareek, Mariusz Pierzchała

**Affiliations:** 1 Department of Genomics and Biodiversity, Institute of Genetics and Animal Biotechnology of the Polish Academy of Sciences, Postępu St. 36A, 05-552 Jastrzębiec, Poland; k.chalaskiewicz@igbzpan.pl (K.C.); k.kepka@igbzpan.pl (K.K.-B.); m.ogluszka@igbzpan.pl (M.O.); e.polawska@igbzpan.pl (E.P.); m.pierzchala@igbzpan.pl (M.P.); 2 Department of Molecular Biology, Institute of Genetics and Animal Biotechnology of the Polish Academy of Sciences, Postępu St. 36A, 05-552 Jastrzębiec, Poland; 3 Industrial Chemistry Institute, Łukasiewicz Research Network, Rydygiera St. 8, 01-793 Warsaw, Poland; mateusz.borkowski@ichp.lukasiewicz.gov.pl; 4 Department of Physiology, Cytobiology and Proteomics, West Pomeranian University of Technology, K. Janickiego St. 29, 71-270 Szczecin, Poland; adam.lepczynski@zut.edu.pl (A.L.); elzbieta_lichwiarska@zut.edu.pl (E.L.); 5 Department of Infectious, Invasive Diseases and Veterinary Administration, Institute of Veterinary Medicine, Faculty of Biological and Veterinary Sciences, Nicolaus Copernicus University, Lwowska St. 1, 87-100 Toruń, Poland; ssharna21@gmail.com (S.S.); vetgarimakalra@gmail.com (G.K.); purohitnihal621@gmail.com (N.P.); pareekcs@umk.pl (C.S.P.)

**Keywords:** aflatoxin B1, gut microbiota, gut–brain axis

## Abstract

Aflatoxins, toxic secondary metabolites produced by *Aspergillus* species, are widespread contaminants in food and feed, with aflatoxin B1 (AFB1) recognized as the most potent carcinogen. Climate change increases the risk of contamination by promoting fungal proliferation. While the hepatotoxic and enterotoxic effects of aflatoxins are well established, emerging evidence highlights their immunosuppressive and neurotoxic potential. Notably, AFB1 disrupts gut microbiota, compromises intestinal barrier integrity, and induces neuroinflammation via the microbiota–gut–brain axis. Probiotics have shown promise in mitigating these effects by modulating microbial balance, enhancing barrier function, and reducing neuroinflammatory responses. This review summarizes current findings on the systemic toxicity of aflatoxins—particularly their impact on the gut–brain axis—and evaluates the therapeutic potential of probiotics in counteracting aflatoxin-induced damage.

## 1. Introduction

### 1.1. General Background

The discovery of aflatoxins dates back to the early 1960s, when the toxins were first crystallized and characterized using thin-layer chromatography. The first documented outbreak, known as “turkey X disease,” resulted in significant mortality among turkeys. It was later determined that the affected birds had been fed Brazilian groundnut meal contaminated with aflatoxins. These compounds were subsequently identified as secondary metabolites produced by *Aspergillus flavus*, a fungus commonly found in soil and on crops grown under warm and humid conditions [1,2].

Although *Aspergillus* thrives in moist environments, aflatoxin production often peaks during drought or dry periods following wet seasons. Mycotoxins, particularly aflatoxins, represent a major threat to agriculture and animal husbandry. It is estimated that approximately 4.5 billion people worldwide—primarily in developing countries—are chronically exposed to aflatoxins. However, low-level exposure also occurs globally through contaminated food, including in industrialized nations [1,2].

According to the European Food Safety Authority [3], the highest concentrations of aflatoxin B1 have been found in legumes, nuts, and oilseeds, particularly in peanuts, pistachios, and related products. In contrast, aflatoxin M1 (AFM1), a hydroxylated metabolite of AFB1 found in milk, is most frequently detected in dairy products.

Aflatoxins also exert substantial effects on livestock, especially pigs, poultry, and cattle [4]. The increasing contamination of cereal grains, the main components of animal feed, with aflatoxins leads to decreased productivity (in milk, eggs, and meat), increased incidence of disease, and organ damage. These outcomes ultimately compromise food quality and security for humans. This issue is particularly acute in developing countries. For instance, in 2013, Uganda lost the opportunity to export 5 million metric tons of maize due to aflatoxin contamination [5].

### 1.2. Literature Overview

Mycotoxins are classified into five major groups based on their chemical structure: fumonisins, aflatoxins, ochratoxins, zearalenone, and trichothecenes. The fungi responsible for their production are typically divided into two categories: field fungi, which grow before harvest, and storage fungi, which proliferate post-harvest [4]. Aflatoxins are secondary metabolites produced primarily by *Aspergillus flavus*, *A. nomius*, *A. parasiticus*, *A. pseudotamarii*, *A. parvisclerotigenus*, and *A. bombycis* [6]. In their pure form, aflatoxins crystallize into colorless to pale yellow solids that are soluble in polar solvents. They are sensitive to ultraviolet light, oxygen, extreme pH values (<3 or >10), and oxidizing agents [4]. The problem of aflatoxin contamination is strongly linked to the One Health concept, which emphasizes the interconnectedness of human, animal, and environmental health [7]. Aflatoxins pose a dual threat: they compromise human health through contaminated food and feed, and they impair animal health and productivity [8]. This duality highlights the need for cross-sectoral collaboration involving agriculture, food safety, veterinary medicine, and environmental stewardship.

Recognizing the wide-reaching consequences of aflatoxin contamination for public health, food systems, and economic stability, the One Health framework promotes integrated mitigation strategies. These include improved agricultural practices, enhanced food safety protocols [9], and environmental interventions to limit fungal proliferation—particularly in the context of climate change [10]. A holistic response to aflatoxin exposure not only safeguards human and animal health but also supports environmental sustainability in line with One Health principles.

To date, more than 20 types of aflatoxins have been identified, with the most extensively studied forms including B1, B2, G1, G2, M1, M2, aflatoxicol, and aflatoxin Q1. AFB1 is the most potent foodborne carcinogenic mycotoxin. It is naturally produced by several Aspergillus species, including *A. flavus*, *A. parasiticus*, *A. nomius*, *A. bombycis*, *A. arachidicola*, *A. minisclerotigenes*, *A. ochraceoroseus*, *A. pseudonomiae*, and *A. rambellii*. The median lethal dose (LD_50_) of AFB1 varies widely by species and sex, ranging from 9000 to 60,000 µg/kg body weight [4].

Aflatoxin B2 is produced by *A. flavus*, *A. arachidicola*, *A. minisclerotigenes*, *A. nomius*, and *A. parasiticus*. Aflatoxins G1 (AFG1) and G2 (AFG2) are synthesized by soil fungi, including *A. parasiticus*, *A. nomius*, *A. bombycis*, *A. arachidicola*, and *A. flavus* [4].

Aflatoxins M1 and M2 (AFM2) are hydroxylated metabolites of AFB1 and AFB2, respectively, formed in mammalian livers. Approximately 1–6% of ingested AFB1 is metabolized to AFM1. The maximum permissible level of AFM1 in milk and dairy products is 0.05 µg/kg in both Europe and the United States [11].

Aflatoxicol, another metabolite of AFB1, is produced through enzymatic reduction by fungi such as *Tetrahymena pyriformis*, *Trichoderma viride*, *Dactylium dendroides*, *Streptococcus lactis*, *Absidia repens*, *Mucor griseocyanus*, *Aspergillus niger*, *Mucor ambiguus*, and *Rhizopus* spp. AFB1 is also metabolized in the microsomal fraction of the liver to yield its monohydroxylated derivative, aflatoxin Q1 [4]. The relative toxicity and mutagenicity of aflatoxins are generally ranked as follows: AFB1 > AFG1 > AFB2 > AFG2 [12].

Aflatoxins are metabolized in the body through phase I and phase II biotransformation processes. In phase I, cytochrome P450 (CYP450) enzymes primarily located in the liver convert aflatoxin B1 via epoxidation, hydroxylation, hydration, and O-demethylation reactions. These enzymatic modifications generate several metabolites, including AFB1-exo-8,9-epoxide (AFBO), AFB2a, AFQ1, AFP1, and AFM1 [6]. In phase II metabolism, these reactive intermediates undergo conjugation with glutathione, a detoxification process that facilitates their excretion via urine and bile. Among these metabolites, AFBO is considered the most genotoxic. It readily forms covalent adducts with DNA, particularly at the N7 position of guanine bases, resulting in AFB1–DNA adducts. These lesions disrupt DNA replication and transcription processes, ultimately causing DNA strand breaks and mutations and contributing to carcinogenesis and mutagenesis [13].

Aflatoxin contamination in food and crops leads to substantial economic losses for both producers and traders. According to a World Health Organization (WHO) report (2018), mycotoxin contamination is responsible for the destruction of approximately 25% of global crop production [14]. The increasing prevalence of foodborne mycotoxins poses serious risks not only to consumer health but also to environmental quality, including potential degradation of soil and groundwater systems [2].

Studies have shown that crops such as sugarcane and groundnut seedlings can absorb aflatoxins from contaminated soil through their roots and accumulate them in aerial tissues [15].

Under natural conditions, concentrations of AFB1 and its metabolites in soil are typically low, owing to the activity of microbial communities capable of degrading these compounds. Microbial consortia play a key role in aflatoxin detoxification, including *Actinobacteria* such as *Nocardia corynebacteroides*, *Mycobacterium fluoranthenivorans*, *Corynebacterium rubrum*, and *Rhodococcus erythropolis*; *α*- and β-*proteobacteria*; and several *Bacillus* species, including *B. thuringiensis*, *B. mycoides*, *B. subtilis*, *B. amyloliquefaciens*, and *B. weihenstephanensis* [16].

As abiotic degradation of aflatoxins primarily affects the soil surface, these toxins may still persist in deeper layers. Microbial degradation leads to a contrasting dissipation time (DT_50_) of 19–23 days in sandy soils and 35–48 days in clay soils. The slower degradation in clay soils is attributed to the reduced bioavailability of aflatoxins due to their higher mineral content [17].

Soil-dwelling organisms such as microbes, earthworms, and nematodes can be adversely affected by aflatoxins [14]. *Caenorhabditis elegans*, a non-parasitic nematode commonly found in soil, is widely used as a model organism for studying the toxicological effects of aflatoxins. Experimental studies have shown that exposure to aflatoxin B1 reduces growth, reproduction, and lifespan in *C. elegans* [18].

Aflatoxins also impact other soil fauna. In *Eisenia fetida*, a species of earthworm, AFB1 exposure leads to visible toxicity symptoms including curling, squirming, excessive mucus secretion, and dehydration. Aflatoxins may also damage earthworm eggs. Under dry conditions and at high doses (e.g., 100 µg/kg), increased genotoxicity has been observed [12]. Environmental factors such as humidity significantly influence the toxicity and degradation rate of AFB1 in soil. Aflatoxins degrade more slowly in dry soil, and interestingly, some soil organisms may contribute to their mitigation. Earthworms such as *Eisenia andrei* can reduce aflatoxin concentrations by ingesting mold-contaminated material [19]. In grain ecosystems, AFB1 negatively affects development and increases mortality in beetles. Specifically, *Ahasverus advena* (Waltl) exposed to 1,000,000 µg/kg AFB1 exhibited delayed larval development and reduced survival. Nevertheless, *A. advena* shows a notably higher tolerance to mycotoxins compared to other insect species such as *Drosophila melanogaster*, *Trichoplusia ni*, *Apis mellifera*, and *Spodoptera littoralis*, which exhibit up to a 100-fold greater sensitivity to aflatoxins [20].

Climate change has a significant impact on aflatoxin production by fungi [14,21,22]. For example, a severe drought in Serbia in 2012 led to a 69% contamination rate of maize with aflatoxins [23]. *Aspergillus* species are known to thrive at temperatures above 20 °C, with optimal aflatoxin production occurring between 25 and 37 °C [14]. Crops most susceptible to aflatoxin-producing fungi are primarily cultivated in tropical and subtropical regions, including large parts of Africa, Asia, and South America [14,21,22]. Although *Aspergillus* species grow best under warm and humid conditions, aflatoxin B1’s production is often intensified by drought and heat stress. This enhancement has been linked to increased expression of aflatoxin biosynthesis genes, such as *aflD* and *aflR* [23]. A 2 °C rise in average temperature could significantly expand the geographical range of aflatoxin contamination in Europe. Predicted hotspots include Greece, Southern Italy (including Corsica and Sardinia), and the Iberian Peninsula, according to climate modeling [21].

While the hepatotoxicity and immunotoxicity of aflatoxins are well documented, emerging research has linked them to gut dysbiosis, intestinal inflammation, and neurological impairment via the MGBA. Still, the clinical translation of these findings is limited, with most evidence derived from animal models.

### 1.3. Study Rationale

Addressing aflatoxin contamination in both food and animal feed is critical to ensuring agricultural sustainability and protecting public health. Soil contamination with aflatoxins may occur when tainted food products are inadvertently returned to the soil during planting, thereby affecting soil quality. These toxins can be absorbed by plant roots, stems, and leaves, subsequently impacting the health of birds and insects that interact with or consume the contaminated vegetation [14]. The systemic effects of aflatoxins extend beyond the liver and digestive tract, involving immune and nervous systems. However, the mechanisms underlying these extrahepatic effects, and the potential for probiotics to counteract them via the gut–brain axis, remain incompletely understood.

### 1.4. Objectives and Method of Review

This review aims to summarize current findings on the systemic toxicity of aflatoxins—particularly their impact on the gut–brain axis—and to evaluate the therapeutic potential of probiotics in counteracting aflatoxin-induced damage. Literature was identified through database searches (PubMed, Scopus, Web of Science) using combinations of the keywords “aflatoxin B1”, “gut microbiota”, “gut–brain axis”, “probiotics”, “immune system”, “neurotoxicity” and “AFB1 detection”. Priority was given to peer-reviewed articles published in the last 5 years, though seminal earlier studies were also included.

### 1.5. Significance

Understanding the interplay between aflatoxins, gut microbiota, and the gut–brain axis could inform strategies to reduce the health burden of mycotoxin exposure. By integrating toxicology, microbiology, and neuroscience perspectives, this review may help guide the development of dietary or microbial interventions such as probiotic supplementation to mitigate aflatoxin-related health risks in both human and veterinary contexts.

## 2. Regulatory Framework

An important consideration in aflatoxin regulation is the variation in acceptable concentration limits across regions, particularly in animal feed. These standards have direct implications for human health, as aflatoxins can accumulate in animal-derived products consumed by people. In animals exposed to contaminated feed, aflatoxin metabolites can be detected in tissues, milk, and eggs [24].

Within the European Union, maximum permissible levels for aflatoxin B1 in feed are specified under Directive 2002/32/EC and are based on a moisture content of 12%:

- Feed materials: 20 μg/kg;
- Complementary and complete feed mixtures: 10 μg/kg;
- Feed mixtures for dairy cattle and calves, dairy sheep and lambs, dairy goats and kids, piglets, and young poultry: 5 μg/kg;
- General feed mixtures for other categories not specified above: 20 μg/kg [25].

In comparison, aflatoxin tolerance levels established by the U.S. Food and Drug Administration (FDA) are presented in Table 1.

Globally, regions such as Africa, the Middle East, South Asia, and parts of Southern Europe are characterized by elevated aflatoxin contamination levels. China, one of the countries most affected by mycotoxins, has recently implemented more stringent regulations governing allowable mycotoxin levels in feed and raw materials [27].

Chinese standards are differentiated based on animal species, age group, production purpose, and the type of feed component involved (see Table 2). The incidence of aflatoxins in feed samples collected in China in 2021 was relatively low [27].

The climatic conditions in Brazil—one of the world’s leading agricultural producers—favor the proliferation of toxic fungal species and promote mycotoxin production [24]. In 2011, the Brazilian Ministry of Agriculture issued an executive order setting the maximum allowable concentrations of aflatoxins B1, B2, G1, and G2 in raw materials for feed production at 50 µg/kg. For peanuts and corn, the limit is more stringent, set at 20 µg/kg [28]. However, studies indicate that these standards are frequently exceeded, highlighting the urgent need for effective prevention and decontamination strategies [24].

In India, the Bureau of Indian Standards (BIS) has established a maximum permissible level of aflatoxin B1 at 20 µg/kg for all types of animal feed. Like Brazil, India’s climate is conducive to fungal growth and aflatoxin contamination. Sustainable preventive measures are therefore essential in developing countries to manage crop contamination both pre- and post-harvest, including improved monitoring and intervention during storage and processing (e.g., in corn silage) [29].

The adoption of such practices, along with stricter regulatory limits, could significantly reduce economic losses and enhance the safety of animal-derived products for consumers.

## 3. Effects of Aflatoxins on the Digestive System

Aflatoxins enter mainly via the gastrointestinal tract through contaminated food [30], with intestinal epithelial cells—exposed to higher concentrations than other tissues—being the first point of contact [31]. The intestinal barrier, maintained by tight junctions, restricts paracellular transport and is a critical defense [32]. As the GI tract is the primary exposure site [33], aflatoxins can severely impair its function. AFB1 disrupts barrier integrity and alters gut microbiota composition, favoring pathogenic bacteria [34]. AFB1 is a major etiological factor in liver cancer, linked to 4.6–28.2% of global hepatocellular carcinoma cases [30]. Hepatotoxicity results from CYP450-mediated bioactivation to a reactive epoxide that forms DNA/protein adducts, triggering oxidative stress, apoptosis, inflammation, hepatic congestion, hepatomegaly, and necrosis [35].

In pigs, dietary AFB1 (280–320 µg/kg) reduced nutrients’ digestibility; impaired barrier integrity; increased *Escherichia coli* in the colon; decreased SOD, CAT, GPx, TAC, and tight junction gene expression; elevated 8-OHdG; and induced lipid peroxidation [34,36].

Ruminants show greater resistance than monogastrics, but sheep are relatively susceptible. In sheep, single doses of 1000–20,000 µg/kg caused gut microbiota shifts (↑ *BF311* spp., *Alistipes* spp.); severe liver lesions; altered antioxidant and apoptotic gene expression; elevated TBIL, AST, ALT; and reduced Ca^2+^, ALB, TP [35,37]. In beef cattle, contaminated feed induced reduced appetite; diarrhea; elevated AST, ALT, ALP; and reduced TP, ALB, Ca^2+^ [38].

In poultry, AFB1 (40–1000 µg/kg) upregulated intestinal and hepatic pro-inflammatory genes (TLR2, NOD1, NF-κB p65, iNOS, TNF-α, IL-6, IL-8), downregulated tight junction genes (occludin, claudin-1), increased hepatic MDA, and decreased antioxidant enzymes [39,40,41]. In ducks, AFB1 reduced T-SOD, GPx, TP, ALB, TAC, GST, and CAT and increased hepatic residues [42,43].

In rats, high doses (25–2500 µg/kg) increased serum liver enzymes, oxidative stress markers (MDA, TBARS, H_2_O_2_, NO, 8-OHdG), and pro-apoptotic genes (*Bax*, *Caspase-3*, *p53*), while reducing GSH, GST, SOD, CAT, GPx, GR, TAC, and anti-apoptotic Bcl-2. Histology revealed necrosis, vascular changes, inflammation, and villous degeneration [44,45,46,47,48].

Mice, though relatively resistant, showed significant effects at certain doses. AFB2 (20 µg/kg) reduced liver mass and GSH and increased ALT, AST, MDA [49]. AFB1 (300 µg/kg) induced intestinal villous atrophy, tight junction protein loss, and apoptosis [50]. Combined AFB1 + AFM1 exposure impaired intestinal integrity, reduced villus height, altered claudin-1/ZO-1, and changed crypt depth [51]. In BALB/c mice (200 µg/kg), AFB1 shortened colon length, thinned mucus, reduced goblet cells, and downregulated antioxidant proteins (Nrf2, NQO1, HO-1), tight junctions (ZO-1, occludin, claudin-1), and microbiota diversity, while increasing pro-inflammatory cytokines and NF-κB activation [52]. In broilers (500 µg/kg), VH and VSA decreased, CD increased, and tight junction gene expression declined [53]. In C57BL/6 mice (300–750 µg/kg), AFB1 reduced liver mass; increased hepatic ROS, MDA, serum/hepatic LPS, pro-inflammatory cytokines, and pyroptosis gene expression; and impaired intestinal barrier and microbiota composition (↓ *Firmicutes*, *Lactobacillaceae*; ↑ *Bacteroidetes*) [54,55].

Rabbits (300 µg/kg) exhibited elevated ALT, AST, ALP, MDA, 8-OHdG and a reduced CAT, GSH, and hepatic AFB1 accumulation (13 µg/kg) [56]. In zebrafish, AFB1 induced intestinal inflammation, villus shortening, mucin loss, tight junction disruption, microbiota shifts, transcriptomic changes in steroid biosynthesis, PPAR signaling, ferroptosis, immune pathways, hepatocyte loss, and oxidative stress-driven gene dysregulation [57].

**Figure 1:**
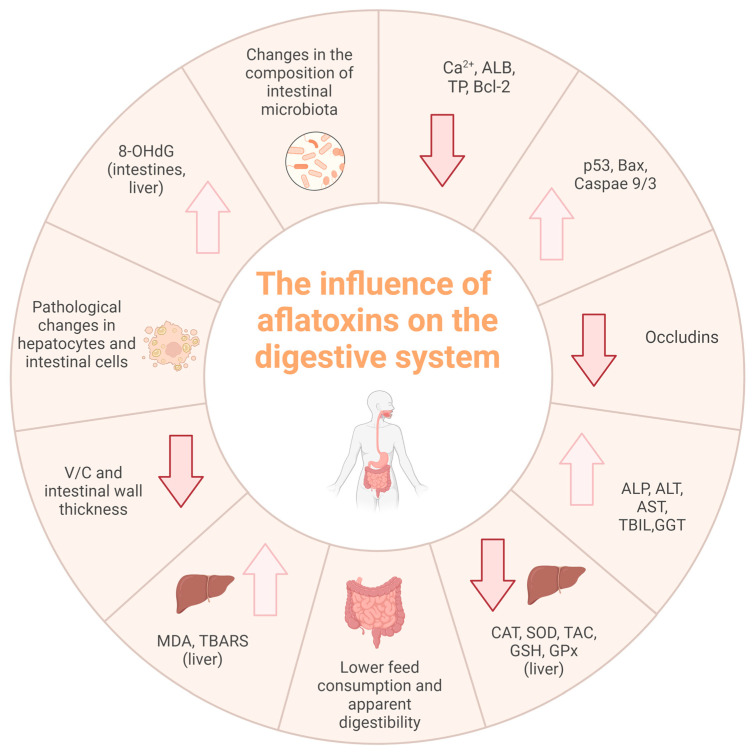
Summary of the major effects of aflatoxins on the digestive system. Aflatoxins impair gut integrity, induce oxidative stress, disrupt tight junctions and microbiota composition, and alter enzymatic and biochemical markers in both the intestines and liver. Upward arrows indicate increased levels; downward arrows indicate reductions. Created in https://BioRender.com.

In conclusion, aflatoxins consistently damage digestive systems across species, with varying susceptibility but well-documented outcomes. Figure 1 summarizes key gastrointestinal effects, underscoring the need for strict monitoring and control in feed and the environment to protect animal health.

In humans, chronic dietary exposure to AFB1 is strongly associated with hepatocellular carcinoma (HCC), especially in regions with high aflatoxin contamination and a concurrent prevalence of hepatitis B virus (HBV). Synergistic interactions between AFB1 and HBV infection markedly increase the risk of HCC, with epidemiological studies estimating that AFB1 exposure accounts for up to 28% of global HCC cases [30]. Clinical manifestations of aflatoxicosis may include jaundice, abdominal pain, hepatomegaly, and, in severe cases, acute liver failure.

Biomarkers of exposure and effect are critical for risk assessment and monitoring. Commonly used biomarkers include serum aflatoxin–albumin adducts (AFB1–lysine), which reflect recent exposure, and urinary AFM1, which indicates metabolic conversion and excretion. Elevated serum transaminases (ALT, AST), bilirubin, and gamma-glutamyltransferase (GGT) are indicative of hepatocellular injury, while decreased albumin and prothrombin time prolongation may signal impaired hepatic synthetic function.

Beyond liver pathology, AFB1-induced intestinal barrier dysfunction and systemic inflammation may contribute to extrahepatic outcomes, including immune dysregulation, increased susceptibility to infections, and potential neurological effects via gut–brain axis perturbation. These clinical implications underscore the importance of early detection of aflatoxin exposure, public health interventions to reduce contamination, and surveillance programs targeting high-risk populations.

## 4. Effect of Aflatoxins on the Immune System

AFB1 disrupts immune homeostasis via concurrent pro-inflammatory and immunosuppressive effects, lowering infection resistance and impairing vaccines’ efficacy. In human microglial cells, AFB1 upregulated pro-inflammatory genes (*TLR2*, *TLR4*, *MyD88*, *iNOS*, *IκB*, *NFκB*, *CXCR4*, *CCR8*), increased IFN-γ and GM-CSF secretion, and activated caspase-3/7, indicating apoptosis [58]. It accelerated dendritic cell maturation but impaired monocyte-derived dendritic cell responsiveness [59]. Dietary exposure in children reduced vaccine responses (↓ IL-4, IL-6, IL-8; ↑ TNF-α) [60], while in HIV-positive patients, AFB1 inversely correlated with CD4^+^ lymphocyte counts [61]. In healthy young adults, naturally occurring AFB1 induced lymphocyte apoptosis, reduced ATP, and increased caspase-3/7 activity [62].

In mice, dietary AFM1 (50 µg/kg) suppressed splenocyte proliferation (PHA, LPS), reduced IFN-γ-producing cells, impaired phagocytosis, decreased IgG, increased IL-10, and lowered CD3^+^, CD4^+^, CD8^+^, and CD19^+^ lymphocyte subsets [63]. Oral AFB1 (450 µg/kg, 21 days) elevated serum IL-1β and IL-6, with hepatic IL-1β, IL-6, and TNF-α upregulation [64]. AFB1 (300 µg/kg, 28 days) increased hepatic cytokines, pro-apoptotic markers (Bax/Bcl-2, Cleaved-caspase-3), and *Keap1* expression and decreased *Nrf2* in the colon; IL-1β, IL-6, and TNF-α were reduced [55].

In vitro, RAW264.7 macrophages exposed to AFB1 showed oxidative stress and increased NOS2, TNF-α, and CXCL2, activating *PPAR*, NF-κB, PI3K-Akt, mTOR, and MAPK pathways [65]. In porcine alveolar macrophages, AFB1 elevated Ca^2+^ and altered lncRNA–miRNA–mRNA networks affecting *CACNA1S*, *RYR3*, and *PRKCG*, causing G1 arrest [66].

In poultry, low-dose/short-term AFB1 exposure stimulated the alternative complement pathway, while high-dose/prolonged exposure suppressed it [67]. Dietary AFB1 (300 µg/kg) increased jejunal apoptosis (↑ Bax, caspase-3; ↓ Bcl-2) [68]. Intraperitoneal AFB1 (1000 µg/kg, 35 days) damaged thymus, spleen, and bursa of Fabricius [69], and oral AFB1 (450 µg/kg, 21 days) elevated hepatic IL-1β, IL-6, IFN-γ, and iNOS [70]. In quail [71] and broilers [40], AFB1 reduced leukocytes, weakened humoral immunity, and increased IL-6 and IL-1β.

**Figure 2:**
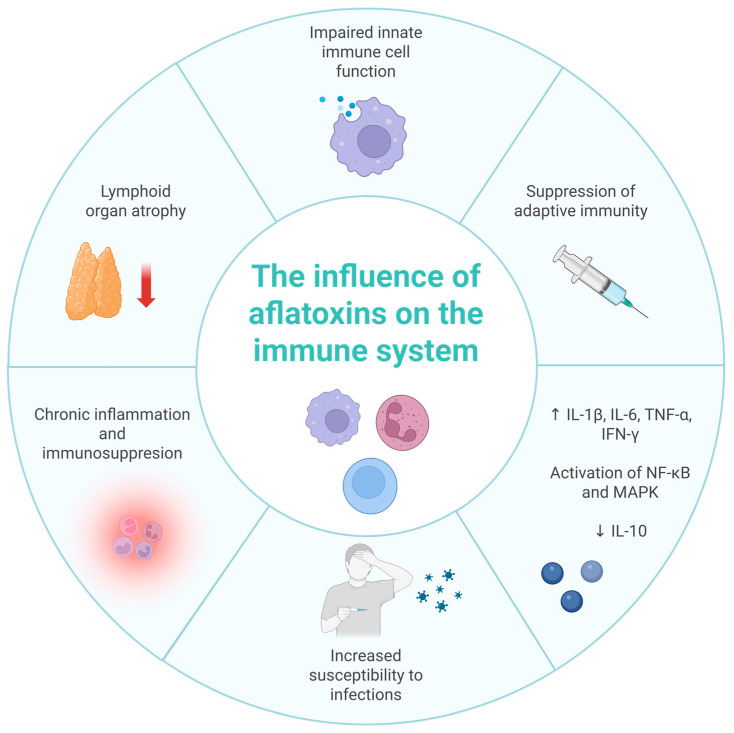
Summary of the immunotoxic effects of aflatoxins. Exposure to aflatoxins can lead to immune dysregulation, impaired response to vaccination, upregulation of pro-inflammatory cytokines and genes, inflammation, and overall immunosuppression. These alterations weaken host resistance and increase susceptibility to infections. Note: ↑ indicates increased expression or activity; ↓ indicates decreased expression or activity. Created in https://BioRender.com.

In juvenile white amur (*Ctenopharyngodon idella*), AFB1 caused spleen and head kidney damage via *Keap1a* upregulation, *Nrf2* suppression, reduced antioxidant activity, p38 MAPK-mediated apoptosis, MLCK activation, and tight junction disruption [72]. Immune effects included complement (C3, C4), IgM, lysozyme, and ACP increased in skin but decreased in spleen/head kidney, along with downregulated antimicrobial peptides (β-defensin-1, LEAP-2A/2B, Mucin2) and upregulated pro-inflammatory cytokines, linked to NF-κB activation and TOR inhibition [73]. In *Litopenaeus vannamei*, dietary AFB1 (5000 µg/kg) induced 12,014 DEGs in the pancreas and 1387 in the intestine, affecting Toll, IMD, proPO, Rab, and GST immune pathways [74]. In *Caenorhabditis elegans*, AFB1 (312–1561 µg/L, 72 h) impaired immunity to *Pseudomonas aeruginosa*, with 312 µg/L as LOAEL, via inhibition of *SKN-1* (*Nrf2* homolog) nuclear translocation [75]. Figure 2 summarizes the key pathways through which AFB1 affects immune function in both humans and animals.

The immunotoxic effects of AFB1 in humans have implications for increased susceptibility to infectious diseases, diminished vaccine efficacy, and worsened prognosis in immunocompromised individuals. Biomarkers such as altered cytokine profiles, reduced lymphocyte counts, and specific gene expression changes could aid in early detection of AFB1-induced immune impairment. Given the overlap of inflammatory and immunosuppressive mechanisms, individuals with chronic viral infections (e.g., HIV, HBV) or undergoing immunosuppressive therapy may be particularly vulnerable.

## 5. Overview of Gut Microbiota Types and Role in Immune Homeostasis and Inflammation Control

The gut microbiota comprises approximately 100 trillion bacterial cells, which is 10 times the total number of human cells [76] The human gut harbors a diverse microbial community dominated by bacteria from the phyla *Firmicutes* (e.g., *Lactobacillus*, *Clostridium*, *Faecalibacterium*) and *Bacteroidetes* (e.g., *Bacteroides*, *Prevotella*), with notable participation from *Actinobacteria* (*Bifidobacterium* spp.), *Proteobacteria* (e.g., *Escherichia* spp., typically in low abundance in healthy conditions), and *Verrucomicrobia* (*Akkermansia muciniphila*, important for mucin degradation and mucosal renewal) [77,78].

These microbes fulfill ecological roles across the gastrointestinal tract, offering a regional specificity that influences metabolic and immunological microenvironments. Their metabolites, especially short-chain fatty acids (SCFAs)—including acetate, propionate, and butyrate—are pivotal immune modulators. SCFAs engage G-protein-coupled receptors (e.g., GPR41, GPR43), inhibit histone deacetylases (HDACs), and epigenetically regulate immune genes to promote anti-inflammatory responses [79,80]. SCFAs reinforce mucosal barrier function by enhancing mucin production, tight junction integrity, and secretory IgA secretion. Commensals like *Bacteroides fragilis* produce immune-modulating polysaccharides (e.g., PSA), promoting regulatory T-cell (Treg) induction and anti-inflammatory cytokine production (e.g., IL-10). Microbial balance suppresses pro-inflammatory pathways, such as NF-κB activation, by limiting exposure to lipopolysaccharide (LPS) from pathogenic bacteria [77,81].

Dysbiosis—characterized by decreased microbial diversity, loss of SCFA-producing species, and overgrowth of *Proteobacteria*—has been linked to chronic low-grade inflammation and immune dysregulation. Such imbalances are implicated in metabolic syndrome, autoimmunity, neurodegeneration, and gastrointestinal disorders. In the context of toxin exposure (e.g., aflatoxin B1), dysbiosis exacerbates gut barrier permeability, systemic inflammation, and neurotoxicity via the gut–brain axis [76,82,83].

## 6. Neurotoxicity of Aflatoxin B1

Mycotoxins, including aflatoxin B1, possess several critical biochemical properties—a high lipophilicity, low molecular weight, and substantial resistance to environmental factors such as elevated temperatures and physicochemical processing [84]. These attributes have raised concerns about the potential impact of AFB1 on both the central and peripheral nervous systems. Although research on aflatoxin-induced neurotoxicity is expanding, current knowledge remains limited and incomplete.

Aflatoxin B1 is a potent inducer of oxidative stress, triggering the overproduction of ROS, lipid peroxidation, and damage to proteins and DNA [85]. These effects disrupt neuronal redox homeostasis and contribute to neurodegeneration. AFB1 induces both mitochondrial-dependent apoptosis and necroptosis, the latter mediated via the RIP1–RIP3–MLKL signaling pathway [86]. It also alters the expression of key apoptotic regulators, including Bax, Bcl-2, and caspases [87]. The *Nrf2–Keap1* pathway, which regulates antioxidant enzymes such as HO-1 and NQO1, is suppressed by AFB1, increasing neuronal susceptibility to oxidative injury. An elevated Bax/Bcl-2 ratio further indicates a shift toward pro-apoptotic signaling and mitochondrial dysfunction.

AFB1 can cross the blood–brain barrier (BBB), leading to endothelial damage and reduced expression of tight junction proteins such as claudin-5 and ZO-1 [88]. This increases BBB permeability and promotes neuroinflammation. In parallel, enhanced production of pro-inflammatory mediators further exacerbates neuronal damage. Neurochemical disruption is also evident.

AFB1 alters the activity of key enzymes such as acetylcholinesterase (AChE) and monoamine oxidase (MAO), contributing to cholinergic dysfunction and cognitive impairment [89]. The hippocampus—particularly the CA1, CA3, and dentate gyrus (DG) regions—is especially vulnerable, exhibiting neuronal degeneration, reduced cell numbers, and impaired synaptic plasticity [90]. Morphological changes have also been observed in the cerebral cortex, cerebellum, and brainstem, including gliosis, demyelination, vascular congestion, and microglial activation [91,92].

Animal model studies aim to elucidate the neurotoxic mechanisms of AFB1 and its potential links to neurodegenerative diseases such as Parkinson’s and Alzheimer’s disease, as well as mood and behavioral disorders. Long-term exposure to AFB1 caused clear neurotoxic effects in 12-week-old male albino rats. The animals received 72 µg/kg body weight of AFB1 for 42 days. The treatment caused significant alterations in plasma lipid profiles, including increased levels of total cholesterol, triglycerides, low-density lipoprotein (LDL), and very low-density lipoprotein (VLDL), with a concomitant reduction in HDL. Glucose-insulin dysregulation was evident, with elevated plasma glucose, insulin, and HOMA-IR, and decreased HOMA-B values. Inflammatory markers (TNF-α, *IDO*) and oxidative stress parameters were also significantly affected, including increased TBARS, xanthine oxidase (XO), and uric acid (UA), alongside decreased levels of GSH, SOD, GST, and GPx. In the brain, AFB1 exposure elevated the activity of AChE and MAO, indicating cholinergic dysfunction and enhanced oxidative stress. Histological examination revealed neuronal degeneration, vacuolization, and thinning of the granule cell layer in the dentate gyrus of the hippocampus [89].

AFB1 exposure has been shown to induce neuroinflammation and impair dopaminergic signaling in C57BL/6J mice. In one study, mice received 1500 µg/kg of AFB1 in drinking water for 21 days. AFB1 exposure led to microglial activation, as evidenced by increased *Iba-1* expression, and elevated levels of pro-inflammatory cytokines, including Il-1β, MCP-1, CSF-2, and CXCL-10. Reduced *IκBα* levels indicated activation of the NF-κB signaling pathway. Furthermore, AFB1 increased the expression of α-synuclein, while reducing tyrosine hydroxylase (TH) expression and dopamine levels, suggesting dopaminergic neuronal dysfunction consistent with Parkinsonian neurodegeneration [91].

AFB1 exposure has been associated with neurotoxicity and cognitive deficits in juvenile mice. In one study, three-week-old male C57BL/6J mice (19–21 g) received 450 µg/kg body weight of AFB1 per day via gavage for 21 days. Behavioral testing using the Novel Object Recognition (NOR) test revealed reduced exploratory behavior toward novel stimuli, indicating deficits in spatial learning and memory. Immunohistochemical analysis showed a significant reduction in neuronal count within the CA1 and CA3 hippocampal regions. These changes were accompanied by altered expression of apoptotic markers, including increased Cleaved-caspase-3 and Bax and decreased Bcl-2, confirming hippocampal cell death as a likely contributor to the observed cognitive impairment [90].

AFB1-induced neurotoxicity may involve nitric oxide signaling and necroptotic cell death. Eight-week-old male NMRI mice (25–30 g) received intraperitoneal injections of AFB1 at 600 µg/kg body weight for four consecutive days. In the Barnes maze test, AFB1-treated mice showed a significantly longer escape latency and greater total distance traveled, along with reduced time spent in the target area. Molecular analysis revealed increased expression of necroptosis markers RIP1, RIP3, and MLKL. The results suggest that AFB1-induced neurotoxicity involves NO-related mechanisms and activation of the necroptotic cell death pathway [86].

AFB1 exposure may be associated with Alzheimer’s disease (AD)-like neuropathology. In one study, male C57BL/6J mice were given drinking water containing 150 µg/L AFB1 for eight weeks. Cognitive deficits were detected in both Morris water maze and Y-maze tests. Histopathological analysis revealed neuronal damage in the CA3 and DG regions of the hippocampus, along with blood–brain barrier impairment characterized by reduced expression of ZO-1 and claudin-5. Immunostaining confirmed AFB1’s accumulation in hippocampal neurons. Gene expression analyses showed upregulation of AD-related markers (*App*, *Psen1*, *Bace1*, *p-Tau*) and a corresponding protein accumulation. AFB1 also increased oxidative stress by elevating Fe^2+^ and MDA levels, while reducing antioxidant defenses, including SOD, CAT, GPx, and GSH. Additionally, ferroptosis-related changes were observed, with decreased expression of GPx, SLC3A2, and SLC7A11 and increased ACSL4. These findings indicate that AFB1 induces oxidative stress and ferroptosis in the hippocampus, contributing to neuronal damage and cognitive impairment resembling Alzheimer’s disease [88].

The effects of AFB1 on depression-like behaviors were studied in male Sprague Dawley rats (7–8 weeks old, 250–350 g) exposed to 5 or 25 µg/kg body weight of AFB1 for four weeks. Rats in the higher-dose group exhibited reduced body weight and food intake, while relative brain weight increased dose-dependently. Behavioral tests, including the saccharin preference and forced swimming tests, revealed symptoms of anhedonia and prolonged immobility, indicating depressive-like states. These findings suggest that AFB1 induces depression-related behaviors in a dose-dependent manner, potentially through the activation of neurotoxic molecular pathways [93].

Male Wistar albino rats (200–250 g) were exposed to 25 µg/kg body weight of AFB1 for eight weeks. Rats displayed anxiety, cognitive deficits, and depressive behaviors in open field, object recognition, and forced swimming tests. Biochemical analyses revealed decreased levels of GSH and GPx, along with a downward trend in GST and SOD activity. Oxidative and inflammatory markers MDA, IL-6, and TNF-α were elevated. Histopathological evaluation of the cerebral cortex and hippocampus showed neuronal degeneration, vacuolization, congestion, and reduced basophilia. A significant reduction in NeuN-positive neuronal populations was observed, while glial fibrillary acidic protein (GFAP) expression was markedly elevated in both the frontal cortex and hippocampus, indicating astroglial activation [94].

Wistar rats were orally administered 70 µg/kg body weight of AFB1 for four weeks. The animals exhibited locomotor impairments, including reduced movement time, decreased velocity, and shorter travel distance. Motor coordination and agility were also compromised, as shown by increased negative geotaxis and reduced limb responsiveness. Heightened anxiety-related behaviors were evident, including prolonged freezing, increased defecation (bolus fecal pellets), and elevated urination. Exploratory behavior declined, as reflected by a lower density of movement traces and diminished activity on heat maps. Biochemical analyses revealed a significant reduction in AChE activity and antioxidant defenses, including GSH, GPx, GST, CAT, and SOD. Concurrently, inflammatory and oxidative stress markers such as myeloperoxidase (MPO), NO, XO, reactive oxygen and nitrogen species (RONS), and lipid peroxidation (LPO) were elevated. Thyroid-stimulating hormone (TSH) levels were decreased. Apoptotic indicators, including caspase-3 and caspase-9 activity, were significantly increased. Histopathological examination revealed neuronal degeneration, pyknosis, vascular congestion, and neuropil vacuolization in the cerebral cortex, as well as reduced nuclear content in Purkinje cells of the cerebellum [95].

Male Wistar rats (6 weeks old, 180 ± 10 g) were orally administered AFB1 at doses of 150 or 750 µg/kg body weight for 30 days. Rats received oral AFB1 at doses of 150 or 750 µg/kg body weight for 30 days. Antioxidant enzyme activities, including SOD, CAT, and GPx, were significantly decreased, along with reduced GSH levels in both brain regions. Pro-inflammatory markers, including IL-6, NO, and MPO, were elevated. Additionally, increased ATP and AMP hydrolysis and enhanced XO activity were observed, indicating oxidative stress. Behaviorally, rats exposed to AFB1 exhibited cognitive deficits in the Y-maze test and reduced mobility in the forced swim test. Elevated AChE activity in the cerebral cortex and hippocampus was also recorded. Histopathological analysis revealed neuronal degeneration, mild neuropil vacuolization, onset of pyknosis, and vascular congestion in the cerebral cortex. In the hippocampus, neurons showed reduced nuclear material. Notably, expression and enzymatic activity of indoleamine 2,3-dioxygenase 1 (IDO1) were significantly increased in both regions, suggesting AFB1-induced neuroinflammation may be mediated via the IDO1 pathway. Collectively, these findings indicate that AFB1 disrupts the redox balance and promotes neurochemical, structural, and behavioral alterations characteristic of cognitive and inflammatory brain disorders [96].

Wistar rats (200 ± 20 g) were orally administered 75 µg/kg body weight of AFB1 per day for 28 days. A significant reduction in brain mass was observed in AFB1-exposed animals. Behavioral assessments revealed marked impairments in learning and memory, including a reduced percentage of spontaneous alternation in the Y-maze, decreased exploration time with novel objects, and a lower recognition index in the NOR test. Impoverished heat maps and simplified movement trajectories further indicated diminished exploratory activity. In the forced swim test, prolonged immobility suggested depressive-like behavior. Neurochemical analysis showed decreased AChE activity in the prefrontal cortex. Antioxidant defenses were significantly compromised, with reduced levels of SOD, CAT, GPx, GSH, GST, and TSH in both the prefrontal cortex and hippocampus. Concurrently, increased activity of XO, NO, and MPO was recorded, indicating intensified oxidative stress and neuroinflammation in these brain regions. These findings support the conclusion that AFB1 induces cognitive and affective dysfunction through oxido-inflammatory and cholinergic mechanisms in the brain [97].

AFB1 exposure in young goats aged 3–4 months has been associated with severe encephalopathy. Over the course of one month, 68 animals died after presenting symptoms such as anorexia, emaciation, nasal discharge, gait abnormalities, progressive limb weakness, and paralysis. In 15 kids, sudden death occurred without preceding clinical signs. Serum analyses revealed elevated LPO and decreased activities of key antioxidant enzymes, including SOD, CAT, and GPx. The feed consumed by the animals contained 380 µg/kg of AFB1. Histopathological findings included mucosal anemia, joint hemorrhages, and congestion of the meninges and lungs. Neuropathological changes were particularly severe, with evidence of cerebral edema, vascular congestion, demyelination, and neuronal degeneration in the cerebellum, midbrain, and thalamus. Additional findings included vasculitis, gliosis, and neuronophagia. Immunohistochemical staining confirmed intense AFB1 expression in neurons and glial cells, particularly in the brainstem and spinal cord. These results indicate the high neurotoxicity of AFB1 in juvenile animals and suggest that oxidative stress-induced apoptosis contributes significantly to its neuropathogenic mechanisms [92].

The neurobehavioral toxicity of AFB1 was evaluated in zebrafish exposed to either 5 µg/L or 20 µg/L AFB1 for seven days. In behavioral tests, zebrafish exposed to 5 µg/L exhibited increased locomotor activity, as indicated by a greater total distance traveled compared to controls. In contrast, the 20 µg/L group showed a reduced movement distance and shorter activity duration, suggesting dose-dependent effects on motor behavior. Lipidomic analysis of brain tissue identified 1734 lipid species, with major classes including fatty acids, glycerolipids, glycerophospholipids, and sphingolipids. In the 5 µg/L group, 70 lipid species were upregulated, while in the 20 µg/L group, 80 were upregulated. Notably, AFB1 exposure disrupted lipid metabolic pathways, particularly sphingolipid metabolism, fatty acid degradation, and glycerophospholipid turnover, indicating that lipid dysregulation may underlie AFB1-induced neurotoxicity [98].

**Figure 3:**
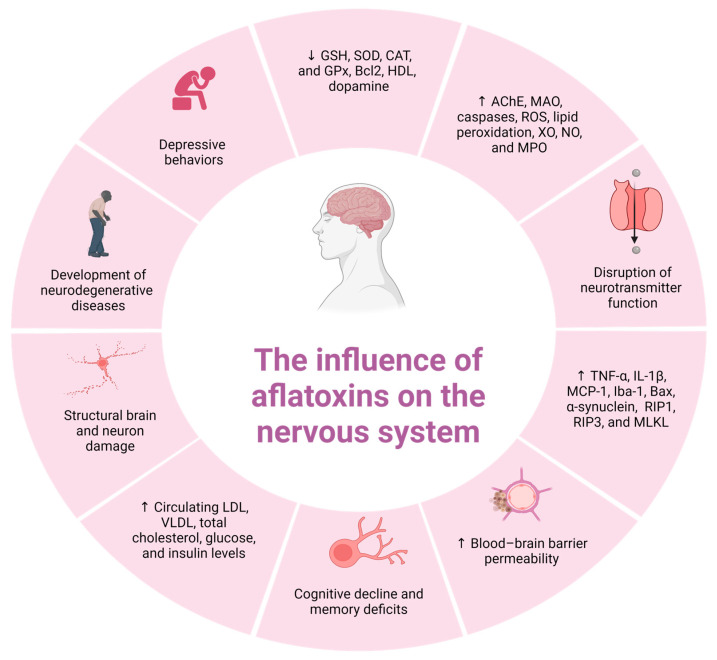
Key effects of aflatoxin B1 on the nervous system. Aflatoxins disrupt blood–brain barrier integrity, impair neurotransmitter balance, induce oxidative stress and neuroinflammation, and affect neuronal structure and behavior. These mechanisms may contribute to cognitive decline, neurodegenerative processes, and psychiatric-like symptoms. Note: ↑ indicates increased expression or activity; ↓ indicates decreased expression or activity. Created in https://BioRender.com.

In summary, AFB1 exerts a marked neurotoxicity in animal models by disrupting lipid metabolism, inducing oxidative stress, promoting apoptosis, impairing neurotransmission, and compromising structural brain integrity. Its detrimental effects on the hippocampus, neurotransmitter systems, and blood–brain barrier establish mechanistic links to neurodegenerative conditions such as Alzheimer’s and Parkinson’s diseases. Continued research is essential to elucidate the molecular pathways underlying AFB1-induced neurotoxicity and to identify reliable biomarkers of exposure and neural damage with translational potential in humans. Figure 3 summarizes the principal mechanisms by which AFB1 affects the nervous system in both humans and animals.

## 7. Microbiota–Gut–Brain Axis and Aflatoxin Neurotoxicity and Protective Effects of Probiotics

In recent years, increasing attention has been directed toward the neuroprotective role of the gut microbiota, with numerous studies supporting bidirectional communication between the gut and the central nervous system (CNS) [99,100,101]. This interaction is encapsulated in the emerging concept of the “gut–microbiota–brain axis,” a dynamic framework that describes the multifaceted communication between the intestinal microbiota and the brain [99,102]. This axis integrates signaling pathways involving the gut microbiota, gastrointestinal tract, immune system, and CNS function [103].

Three principal mechanisms underpin this axis: (1) modulation of autonomic and sensory–motor pathways; (2) immune system activation (approximately 70% of immune cells reside in the gut, capable of influencing brain function via cytokines and inflammatory mediators); and (3) regulation of neuroendocrine signaling [104,105,106]. Key mediators include neuroactive bacterial metabolites such as LPS, microbe-associated molecular patterns (MAMPs), and host- or diet-derived compounds. Bacterial metabolism of dietary amino acids produces neurotransmitters, phenols, and polyamines, while fermentation of polysaccharides generates short-chain fatty acids (SCFAs), notably butyrate. These microbial products can reach the brain through systemic circulation or neural pathways such as the vagus and afferent nerves [107,108]. In the CNS, microbial-derived neurotransmitters contribute to neuronal signaling [109], while SCFAs modulate neuronal receptors, neuroimmune responses, enteroendocrine activity, blood–brain barrier integrity, and epigenetic regulation [110]. Among these, butyrate is considered one of the most potent microbial modulators of neuronal activity [108,111].

Dysbiosis, defined as an imbalance in the composition and function of the gut microbiota, is recognized as a contributing factor in numerous chronic human diseases, including colorectal cancer, diabetes, neurodegenerative and psychiatric disorders, asthma, Crohn’s disease, irritable bowel syndrome, allergies, obesity, eczema, cardiovascular disease, and hepatic encephalopathy [112,113,114,115,116]. It also plays a role in the pathogenesis of neurodevelopmental conditions such as autism spectrum disorder and attention-deficit hyperactivity disorder (ADHD) [108].

Emerging evidence suggests that gut dysbiosis is a key factor in age-related neurodegeneration [101,117]. Disruption of microbial homeostasis is frequently accompanied by increased intestinal permeability, elevated plasma levels of LPS, and compromised BBB integrity [105]. Moreover, dysregulation of the gut microbiota has been implicated in enhanced neurotoxicity following exposure to persistent organic pollutants, including aflatoxins [93,106,108,118].

As previously discussed, oxidative stress and the resulting inflammatory response play a central role in the toxic effects of AFB1 on the digestive, nervous, and immune systems [119]. AFB1 exposure compromises intestinal barrier integrity and increases permeability, thereby enhancing systemic vulnerability to toxic insult [120].

In CNS, AFB1 induces a robust inflammatory response. Increased infiltration of inflammatory cells, particularly neutrophils and macrophages, has been observed in nervous tissue, reflecting immune activation [121]. This is accompanied by activation of glial cells, including microglia and astrocytes, which are key mediators in the progression of various neurodegenerative disorders [122].

Evidence from in vitro studies and animal models suggests that AFB1-induced neurotoxicity is mediated through several physiological disruptions: reduced levels of brain-derived neurotrophic factor (BDNF), impaired neurotransmitter signaling, altered neuropeptide secretion, and neuroimmune suppression [87,122]. These changes may underlie the abnormal behavioral phenotypes and long-term neural dysfunction associated with AFB1 exposure.

Recent studies have emphasized the therapeutic potential of probiotics in disorders associated with dysregulation of the gut–microbiota–brain axis. Probiotics act as immunomodulators by influencing gut-associated lymphoid tissue, which is distributed throughout the gastrointestinal tract [123].

Their primary mechanism of action involves the preservation of intestinal epithelial integrity and function. This is achieved through (a) direct modulation of epithelial cell activity, including stimulation of mucin secretion by goblet cells and increased expression of β-defensins by epithelial cells; (b) enhancement of mucosal immunity by increasing the number of IgA-producing cells; and (c) suppression of pathogenic bacteria through competitive inhibition or downregulation of virulence gene expression [124].

Probiotics may mitigate the toxic effects of AFB1 through several complementary mechanisms. These include enhancement of gut barrier integrity, modulation of the gut microbiota’s composition, and direct detoxification of AFB1. In addition, probiotics have been shown to attenuate AFB1-induced neuroinflammation by downregulating the TLR4/MyD88/NF-κB signaling pathway activated by LPS and by inhibiting microglial activation [108,125].

The effects of a probiotic formulation (ProBiotic-4) containing *Bifidobacterium lactis*, *Lactobacillus casei*, *Bifidobacterium bifidum*, and *Lactobacillus acidophilus* on the gut–microbiota–brain axis and cognitive decline were evaluated in nine-month-old senescence-accelerated prone 8 (SAMP8) mice over a 12-week period. Probiotic treatment significantly improved memory function, reduced neuronal and synaptic damage, and attenuated glial activation. Additionally, alterations in microbiota composition were observed both in fecal and brain samples. ProBiotic-4 attenuated age-associated impairments in intestinal and blood–brain barrier integrity, lowered IL-6 and TNF-α levels at both mRNA and protein levels, reduced circulating and brain LPS concentrations, and inhibited TLR4 expression and NF-κB nuclear translocation in brain tissue. Furthermore, the probiotic reduced markers of DNA damage and oxidative stress, including γ-H2AX and 8-OHdG, and prevented retinoic acid-inducible gene I (RIG-I) multimerization. These findings suggest that modulation of the gut microbiota via probiotics may have therapeutic potential in alleviating age-related cognitive dysfunction [106].

The neuroprotective effects of selected probiotic strains were evaluated in SH-SY5Y neuroblastoma cells exposed to oxidative stress induced by hydrogen peroxide. The investigated strains included *Lactobacillus rhamnosus* GG (commercial), *L. delbrueckii* KU200170, *L. plantarum* KU200661 (both isolated), and *L. lactis* KC24-CM (conditioned medium). HT-29 intestinal epithelial cells were used to generate conditioned media with heat-killed probiotics. Among all the tested strains, *L. lactis* KC24-CM exhibited the most prominent protective effect, as reflected by improved cell viability, an enhanced expression of BDNF, and a reduced Bax/Bcl-2 ratio, indicating attenuation of apoptosis [126].

Exopolysaccharides (EPSs) obtained from lactic acid bacteria have shown antioxidant and neuroprotective properties in SH-SY5Y human neuroblastoma cells exposed to amyloid beta (Aβ)-induced toxicity. EPS treatment preserved antioxidant–oxidant homeostasis by maintaining the activity of SOD, CAT, and GPx. These effects were mechanistically linked to the upregulation of ERK1/2, JNK, JUN, NF-κB/p65, and p38 signaling pathways, alongside downregulation of AKT/protein kinase B. The results suggest that EPS may protect against oxidative stress-induced neurodegeneration [127].

*Lactobacillus plantarum* CRL 1905 showed neuroprotective effects in mouse neuro-2a (N2a) neuroblastoma cells exposed to the mitochondrial toxin 1-methyl-4-phenylpyridinium (MPP^+^). Probiotic treatment improved cell survival and significantly reduced IL-6 secretion, suggesting anti-inflammatory effects [128].

In a related in vitro experiment, intracellular extracts from *Lactiplantibacillus plantarum* CRL2130 reduced oxidative stress and IL-6 release in MPP^+^-treated N2a cells, helping to maintain neuronal viability and lower ROS levels. In an in vivo Parkinson’s disease model, oral administration of *L. plantarum* CRL2130 significantly improved motor function in C57BL/6 mice receiving repeated MPP^+^ injections. Treatment resulted in reduced concentrations of pro-inflammatory cytokines (IL-6, TNF-α, IFN-γ, and MCP-1) and elevated anti-inflammatory IL-10 levels in both serum and brain tissues. These findings indicate that *L. plantarum* CRL2130 exerts robust antioxidant and anti-inflammatory effects and may offer neuroprotection in neurodegenerative disease models [129].

The therapeutic potential of probiotics in reducing neurotoxicity and oxidative stress was investigated in Syrian golden hamsters. The animals were divided into experimental groups receiving either a neurotoxic dose of propionic acid (PPA; 250,000 µg/kg b.w. for 3 days) or a single dose of clindamycin (30,000 µg/kg b.w.). Two additional groups were co-treated with the same neurotoxins followed by probiotic supplementation (200,000 µg/kg b.w./day) for three weeks. The probiotic formulation consisted of *Bifidobacterium infantis*, *B. breve*, *Lactobacillus acidophilus*, *L. bulgaricus*, *L. casei*, *L. rhamnosus*, and *Streptococcus thermophilus* (1 × 10^9^ CFU/g). Neurotoxin exposure caused gut microbiota dysbiosis, notably the emergence of *Clostridium* spp. Probiotic treatment effectively restored microbial balance and eliminated *Clostridium* from the gut. In parallel, both neurotoxin-treated groups exhibited significant reductions in brain GSH levels. Probiotics were more effective in restoring GSH levels in clindamycin-treated animals than in those receiving PPA. Lipid peroxidation was markedly elevated following either PPA or clindamycin exposure; however, probiotic supplementation significantly reduced this effect in the PPA group, with modest results in the clindamycin group. CAT activity increased in both neurotoxin-treated groups compared to controls. Probiotics improved CAT activity more prominently in clindamycin-treated animals than in PPA-treated ones. GST activity was suppressed by both neurotoxins; probiotics partially restored GST activity in the clindamycin group, though levels remained lower than in controls. Creatine kinase activity was elevated across all treated groups, but probiotic supplementation attenuated this elevation. Lactate dehydrogenase (LDH) activity significantly declined only in clindamycin-treated animals; the effect of PPA on LDH was minimal. Overall, the findings suggest that gut dysbiosis induced by neurotoxins such as PPA and clindamycin may contribute to neurotoxicity via the gut–brain axis. Probiotic supplementation showed beneficial effects by rebalancing gut microbiota and modulating oxidative stress markers, highlighting their potential in mitigating toxin-induced neuroinflammation and neurodegeneration [103].

The protective effects of *Lactobacillus plantarum* ATCC8014 against acrylamide (AA)-induced oxidative damage were examined in male Sprague Dawley rats. The animals received lyophilized *L. plantarum* at doses of 10^7^, 10^8^, or 10^9^ CFU/mL (1 mL/day), administered either one hour before or after AA exposure (40 mg/kg b.w./day). Probiotic supplementation enhanced SOD and CAT activity and elevated GSH levels in the hippocampus, cerebellum, and small intestine, while reducing lipid peroxidation. These effects were most pronounced in the high-dose group (10^9^ CFU/mL). Histological analysis revealed minimal neuronal and glial damage in the high-dose group, whereas low-dose groups exhibited partial improvement with residual structural abnormalities. Similarly, intestinal epithelial damage was reduced or absent in the high-dose groups. Preventive application of probiotics was slightly more effective than therapeutic administration. The authors concluded that *L. plantarum* ATCC8014 may reduce AA absorption by enhancing gut barrier function and potentially binding to the toxin, forming complexes that facilitate excretion [130].

In a study involving albino female rats, animals were fed AFB1-contaminated feed (40 µg/kg) for eight weeks. This was followed by a four-week administration of a probiotic preparation derived from lactic acid bacteria. AFB1 exposure disrupted lipid metabolism in the brain, as evidenced by significantly increased concentrations of cholesterol, triglycerides, and phospholipids. This pattern of ectopic lipid accumulation is characteristic of lipotoxicity, which may contribute to the pathogenesis of neurodegenerative conditions such as Parkinson’s disease, Alzheimer’s disease, and Refsum disease. Probiotic treatment significantly reduced lipid concentrations in the brain. In the jejunum, AFB1 caused a marked reduction in cholesterol and triglyceride levels, which were partially normalized by probiotic supplementation. In the ileum, AFB1 decreased triglyceride and phospholipid levels, while probiotics significantly restored phospholipid content [131].

In another study, adult male rats were orally exposed to aflatoxin B1 at a dose of 25 μg/kg body weight per week for eight weeks. At the same time, they received daily supplementation with the multispecies probiotic formulation VSL#3 (2.5 × 10^10^ CFU/day). Probiotic treatment significantly improved brain antioxidant status by increasing GSH, GPx, GST, and SOD levels. It also lowered concentrations of MDA, TNF-α, and IL-6. Behaviorally, rats in the probiotic group showed fewer anxiety-like and depressive-like symptoms compared to those exposed to AFB1 alone. These findings indicate that probiotic supplementation can reduce oxidative damage and neuroinflammation, leading to better neurobehavioral outcomes in AFB1-exposed animals [94].

In a related study, male mice were given 51.8 µL of AFB1 and 30 µL of *Bacillus amyloliquefaciens* B10 daily by oral gavage for 28 days. *B. amyloliquefaciens* B10 markedly attenuated cecal inflammation induced by AFB1. Co-administration resulted in increased expression of tight junction proteins (occludin, claudin-1, and ZO-1) and reduced expression of pro-inflammatory markers (MyD88, TNF-α, IL-6, IL-1β, NF-κB) compared to the AFB1-only group. Moreover, gut microbiota composition was improved, with a significant reduction in *Bacteroides* and *Bacteroidetes* and an increase in *Firmicutes*. These findings support the use of *B. amyloliquefaciens* B10 as a potential feed additive for modulating intestinal inflammation and restoring gut microbial balance in animals exposed to AFB1 [132].

In another study, researchers analyzed the protective effects of a probiotic-based adsorbent (PBA) against AFB1-induced toxicity in *Piaractus mesopotamicus*. For 15 days, fish were fed diets containing either 25 or 400 µg AFB1/kg feed, with or without PBA supplementation. PBA-treated groups showed increased feed intake and, consequently, greater AFB1 consumption. Despite this, the probiotic adsorbent improved digestive enzyme activity—protease activity increased in the stomach and pyloric caeca, and amylase activity was elevated in the intestine, particularly at the lower AFB1 dose. Histopathological assessment revealed that PBA attenuated damage to the gastric microvilli and positively influenced mucosal thickness in the stomach and both anterior and posterior intestinal segments. These results suggest that PBA supports digestive physiology and mitigates AFB1 toxicity in fish [133].

In another study, researchers investigated the impact of *Bifidobacterium breve* on gut–liver axis integrity in C57BL/6J mice subjected to a high-fat diet (HFD) and chronic AFB1 exposure (200 µg/kg b.w./day) for nine weeks. Probiotic treatment (10^7^ CFU/day) significantly reduced weight gain, caloric intake, liver index, and serum markers of liver injury (ALT, AST), lipid dysregulation (TAG, LDL/HDL), and hepatic fat accumulation. Glucose tolerance improved, and histological analysis confirmed reduced hepatic steatosis and better tissue architecture. *B. breve* also modulated lipid metabolism—reducing total fatty acids, triglycerides, and saturated fatty acids, while increasing levels of acetylcarnitine, phosphatidylglycerol, and phosphatidylserine. In the ileum, VH, CD, and ZO-1 expression were elevated. Furthermore, the probiotic increased fecal SCFA concentrations, particularly acetic, isobutyric, and isovaleric acids. It suppressed pro-inflammatory cytokines (TNF-α, IL-6) and elevated anti-inflammatory IL-10 levels. Microbiota analysis showed decreased *Deferribacteres* abundance and improved β-diversity. Metabolomic profiling revealed modulation of 26–45 metabolites involved in β-alanine, butyric acid, and glutathione pathways, as well as key metabolic networks linked to energy and amino acid metabolism. These findings underscore the multifaceted protective role of *B. breve* in counteracting AFB1-induced metabolic and inflammatory disturbances [134].

The reviewed studies indicate that probiotic supplementation helps maintain gastrointestinal mucosal integrity and supports blood–brain barrier function through modulation of the gut microbiota’s composition and related signaling pathways. Enhancing microbial diversity and taxonomic balance through probiotic intervention has been shown to positively influence the gut–brain axis [135]. This modulation reduces the intestinal absorption of neurotoxic agents, thereby limiting their accumulation in neural tissues [108]. Moreover, probiotics may aid in restoring impaired detoxification pathways, correcting dysbiosis, and attenuating oxidative stress. In various toxicity models, administration of a probiotic has effectively reduced pro-oxidant and pro-inflammatory responses, leading to improvements in behavior and emotional regulation, including reduced anxiety- and depression-like symptoms [94].

Although research specifically targeting the protective effects of probiotics against AFB1-induced neurotoxicity remains limited, the available data, particularly regarding their antioxidative and anti-inflammatory actions, highlight a promising therapeutic potential. These findings underscore the need for further investigation into the role of probiotics in mitigating the impact of AFB1 on the microbiota–gut–brain axis. Continued exploration in this area may yield novel strategies for preventing or alleviating the neurotoxic consequences of aflatoxin exposure.

Despite promising findings from animal studies and in vitro experiments, the translation of probiotic-based interventions into clinical or veterinary practice requires careful consideration. Many of the probiotic strains examined, such as *Lactobacillus plantarum*, *Bifidobacterium breve*, and *Bacillus amyloliquefaciens*, demonstrate therapeutic potential; however, their efficacy and safety in humans and livestock remain to be fully validated in large-scale, controlled trials. While some strains are already used in functional foods or feed additives, others remain experimental and may require regulatory approval before clinical application.

Furthermore, the use of probiotics faces several limitations. These include variability in strain-specific effects, lack of standardization in formulation and dosage, and uncertainty regarding their survival and colonization capacity in the gastrointestinal tract. Host-related factors such as age, health status, microbiota composition, and diet can also influence probiotic efficacy. In some cases, unintended consequences such as temporary dysbiosis or immunomodulatory imbalance have been reported, particularly in immunocompromised individuals.

**Figure 4:**
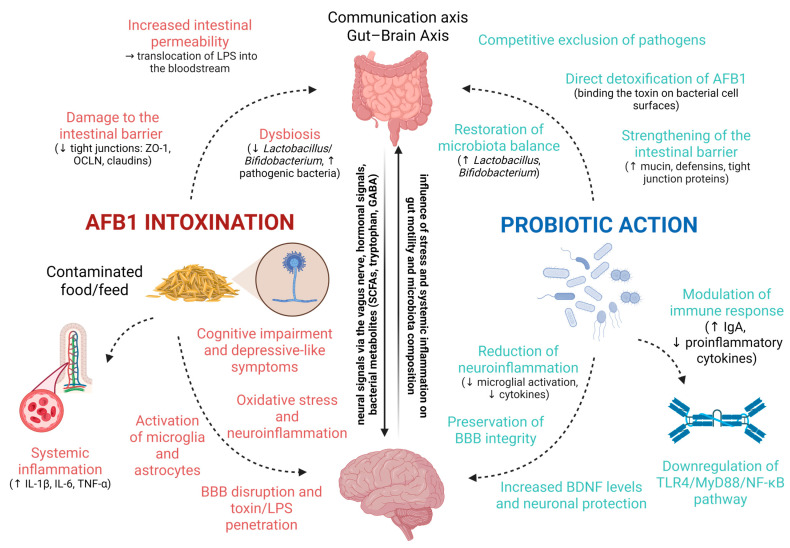
Schematic representation of the gut–brain axis in AFB1 intoxication and probiotic action. AFB1 disrupts intestinal barrier integrity and induces dysbiosis, systemic inflammation and neuroinflammation, whereas probiotics restore microbiota balance, strengthen barrier function, modulate immune responses, detoxify AFB1, and provide neuroprotection. Note: ↑ indicates increased expression or activity; ↓ indicates decreased expression or activity. Created in https://BioRender.com.

Therefore, although probiotics offer a promising avenue for mitigating AFB1 toxicity, further research is necessary to establish their optimal use, long-term safety, and efficacy in both human and veterinary settings. The key interactions between AFB1-induced gut dysbiosis, systemic inflammatory responses, and neurotoxicity, as well as the proposed protective mechanisms of probiotics, are illustrated in Figure 4. This schematic representation summarizes how AFB1 compromises intestinal barrier integrity and promotes dysbiosis, systemic inflammation, and neuroinflammation, while probiotic interventions restore microbial balance, reinforce barrier function, modulate immune responses, detoxify AFB1, and exert neuroprotective effects.

### Therapeutic Potential and Clinical Application

The growing body of experimental evidence highlights the multifaceted role of probiotics in counteracting AFB1-induced toxicity through modulation of the microbiota–gut–brain axis, enhancement of mucosal barrier integrity, reduction in oxidative stress, and attenuation of pro-inflammatory signaling pathways. Strains such as *Lactobacillus plantarum*, *Bifidobacterium breve*, *Bacillus amyloliquefaciens*, and multispecies formulations (e.g., VSL#3 [94]) have shown protective effects in animal and in vitro models, leading to improvements in neurobehavioral outcomes, gut barrier function, and metabolic homeostasis [94,108,132,134]. From a translational perspective, these findings open avenues for clinical and veterinary applications:

- Human health: Probiotic supplementation may serve as an adjunct strategy for populations at high risk of aflatoxin exposure (e.g., in endemic regions), potentially reducing systemic inflammation, protecting liver and neural function, and supporting immune resilience.
- Veterinary practice: In livestock, targeted probiotic feed additives could limit the bioavailability of AFB1, protect gut and liver health, and improve productivity by maintaining nutrient absorption and immune competence. Importantly, in poultry farming, the use of probiotics has also been associated with a reduction in antibiotic consumption, supporting both animal welfare and antimicrobial stewardship.
- Neuroprotection: Given their ability to modulate the gut–brain axis, selected probiotic strains hold promise for mitigating neurotoxicity and preserving cognitive function in both humans and animals.

However, several limitations must be addressed before routine clinical implementation:

- Strain specificity: Probiotic effects are highly strain-dependent, and not all strains confer equal protection against AFB1 toxicity.
- Standardization: Variability in formulation, dosing, and delivery methods complicates reproducibility and cross-study comparisons.
- Colonization efficiency: Survival through the gastrointestinal tract and long-term persistence in the host microbiota remain uncertain for many strains.
- Host factors: Age, health status, baseline microbiota composition, and diet can all influence efficacy.
- Safety considerations: While generally regarded as safe, probiotics may induce transient dysbiosis or unwanted immune modulation in immunocompromised individuals.

To advance probiotic-based interventions from experimental settings to clinical and veterinary practice, large-scale, randomized controlled trials are essential to validate efficacy, determine optimal dosing regimens, and assess long-term safety. Regulatory frameworks will also need to adapt to accommodate probiotics with specific health claims related to aflatoxin mitigation. Given the predominance of preclinical evidence, synthesizing these findings in a structured format allows for clearer visualization of experimental parameters and outcomes. Accordingly, Table 3 summarizes the relevant animal studies, providing a comparative perspective on the probiotic-mediated mitigation of AFB1-induced toxicity through modulation of the microbiota–gut–brain axis.

## 8. Detection of Aflatoxins in Biological Samples

Monitoring human exposure relies on a combination of immunoassay screening and confirmatory chromatographic–mass spectrometric methods tailored to complex matrices such as serum, plasma, and urine. Enzyme-linked immunosorbent assays (ELISAs) remain widely used for high-throughput screening of AFB1, AFM1, and protein adducts in serum/plasma, because they are inexpensive and scalable; however, cross-reactivity with structurally related mycotoxins and matrix effects can yield false positives, so positive screens are typically verified by chromatographic techniques. Rapid lateral flow immunoassays (LFIAs) are increasingly adopted for point-of-care or field triage (e.g., AFM1), offering minute-scale “yes/no” decisions, and recent signal-amplified formats have improved their sensitivity compared with earlier strips [136,137].

For quantification, high-performance liquid chromatography with fluorescence detection (HPLC-FLD) remains a workhorse for AFM1 in urine and milk after cleanup—commonly via immunoaffinity columns or other solid-phase extraction—benefiting from mature workflows and robust performance across laboratories. Because native fluorescence of some aflatoxins is weak, post-column or photochemical derivatization is often used to boost detectability; multi-lab evaluations continue to demonstrate reproducibility for AFB1/AFM1 in urine by HPLC-FLD. Ultra-high-performance LC (UHPLC) shortens run times and, when coupled to tandem mass spectrometry (LC–MS/MS), provides superior selectivity and multi-analyte capability [138,139,140].

LC–MS/MS has become the reference platform for exposure biomonitoring, because it reaches pg/mL concentrations in plasma/serum and urine, resolves co-eluting interferences, and supports broad panels (AFB1, AFM1, AFQ1, AFP1, etc.). Two biomarker classes are particularly informative. First, albumin adducts measured as AFB1–lysine (AFB1-lys) in digested serum proteins integrate exposure over ~2–3 months; recent work optimized reference materials, addressed pre-analytical variables (hemolysis, storage temperature, plasma vs. serum), and validated human serum albumin normalization to reduce variance. Second, urinary biomarkers reflect recent intake (24–48 h), with AFM1 being the most common; highly sensitive LC–MS/MS methods also quantify urinary AFB1–N7-guanine (AFB1-N7-Gua) and related Fapy adducts, providing mechanistic evidence of genotoxic doses and facilitating population studies. Population-scale LC–MS/MS surveys increasingly adopt multiplexed “exposome” panels that include aflatoxins alongside other mycotoxins, enabling seasonal or dietary pattern analyses [141,142,143,144].

Efficient sample preparation is essential to counter matrix suppression. Protein precipitation and solid-phase extraction (SPE)—especially immunoaffinity cleanup for AFM1—remain standard for biofluids. QuEChERS-style procedures, originally developed for foods, have been adapted and validated for multi-mycotoxin LC–MS/MS workflows; while most validations are in foods/feeds, the approach (salt-assisted extraction and dispersive SPE) translates well to biofluids in multi-analyte contexts, provided matrix-matched calibration and stable-isotope internal standards are used [138,145,146].

In summary, a tiered strategy is recommended: (i) immunoassay (ELISA or LFIA) for rapid screening; (ii) HPLC-FLD for routine AFM1 confirmation where MS is unavailable; and (iii) LC–MS/MS for definitive quantification of AFM1 in urine and AFB1-lys in serum (long-term exposure), with optional urinary DNA adducts (AFB1-N7-Gua/FapyGua) to index genotoxic burden. Standardizing pre-analytical handling, applying isotope-labeled internal standards, and reporting creatinine- or albumin-normalized values where appropriate will improve comparability across cohorts and time [141,147]. To facilitate comparison and practical applications, the analytical approaches discussed are compiled in Table 4, which provides a comparative overview of detection methods for aflatoxins and their metabolites, including their principles, target analytes, advantages, and limitations.

## 9. Future Perspectives

Although significant progress has been made in understanding the toxicological mechanisms of AFB1, several critical gaps remain. Firstly, the majority of experimental data is derived from in vitro or animal models, and robust clinical trials are lacking to validate these findings in human populations. Secondly, the interactions between AFB1 exposure, gut microbiota composition, and systemic health require further exploration, particularly in the context of chronic low-dose exposure that more closely mirrors real-world scenarios. Advanced omics technologies such as metagenomics, transcriptomics, and metabolomics offer promising avenues to elucidate host–microbiota–toxin interactions and identify novel biomarkers of exposure and effect. Additionally, the development of probiotic-based interventions, as well as dietary and pharmacological strategies to mitigate AFB1 toxicity, should be pursued with rigorous safety and efficacy evaluations. Regulatory frameworks should also adapt to incorporate biomonitoring data and emerging analytical technologies into risk assessment and public health policies. Ultimately, a multidisciplinary approach combining toxicology, microbiology, nutrition, and epidemiology will be essential to translate preclinical findings into tangible preventive and therapeutic measures.

Another promising direction lies in the advancement of detection methodologies for AFB1 and its metabolites in biological matrices. Although current techniques such as LC–MS/MS, HPLC–FLD, and immunoassays have achieved a high sensitivity and specificity, their application in large-scale epidemiological studies is often limited by cost, throughput, and infrastructure requirements. Future efforts should focus on miniaturized, portable, and point-of-care devices capable of real-time, on-site detection with minimal sample preparation. Integration of novel biosensing platforms such as nanomaterial-based sensors, microfluidic chips, and surface-enhanced Raman spectroscopy could enable rapid, multiplexed analysis of mycotoxins alongside other biomarkers of exposure. In parallel, the combination of targeted and untargeted metabolomics may improve the detection of emerging aflatoxin metabolites and adducts, enhancing our understanding of individual exposure profiles. Harmonization of detection protocols and validation across laboratories will be critical to ensure data comparability and facilitate the incorporation of biomonitoring results into public health surveillance and regulatory decision-making [144].

## 10. Conclusions

Aflatoxin B1 remains a significant global health concern due to its potent hepatotoxic, immunotoxic, and neurotoxic effects, as well as its capacity to disrupt gut microbiota homeostasis. Current evidence underscores the multifaceted pathways through which AFB1 exerts its toxic effects, involving oxidative stress, inflammation, apoptosis, and immune dysregulation. The reviewed studies highlight the potential of probiotics and other microbiota-targeted strategies to mitigate AFB1-induced damage, although their application in clinical or veterinary practice requires further validation. Advances in analytical detection methods now allow for highly sensitive biomonitoring of AFB1 and its metabolites in biological samples, which will be crucial for both epidemiological surveillance and intervention studies. Continued research integrating mechanistic insights with translational applications will be key to reducing the burden of aflatoxin-related diseases in both humans and animals.

## Figures and Tables

**Table 1:** Standards for aflatoxins in animal feed established by the U.S. Food and Drug Administration [26].

AF Level [μg/kg]	Class of Animal	Commodities
20	Dairy animals, animals not specified in other categories, or animals with unknown use	For corn, peanut products, cottonseed meal, and other animal feeds and feed ingredients
20	Immature animals	For corn, peanut products, and other animal feeds and feed ingredients, excluding cottonseed meal
20	Pets of all ages (e.g., dogs, cats, rabbits)	For corn, peanut products, cottonseed meal, other food ingredients, and complete pet food
100	Breeding cattle, breeding swine, and mature poultry (e.g., laying hens)	For corn and peanut products
200	Finishing swine (weighing 100 pounds or more)	For corn and peanut products
300	Beef cattle, swine, and poultry (regardless of age or breeding status)	For cotton seed meal
300	Finishing beef cattle (e.g., feedlot cattle)	For corn and peanut products

**Table 2:** Standards for aflatoxin B1 in animal feed established by the Chinese government [27].

	Feedstuff	AB1 Level [μg/kg]
Raw materials	Maize processing products, peanut meal	≤50
	Vegetable oil	≤10
	Maize oil, peanut oil	≤20
	Other plant-based feed materials	≤30
Products	Concentrated feed for piglets and young birds	≤10
	Concentrated feed for meat ducks, growing ducks, and ducks for egg production	≤15
	Other concentrated feed	≤20
	Calf and lamb concentrate supplement	≤20
	Concentrate supplement for lactation	≤10
	Other concentrate supplements	≤30
	Compound feed for piglets and young birds	≤10
	Compound feed for meat ducks, growing ducks, and laying ducks	≤15
	Other formula feed	≤20

**Table 3:** Summary of preclinical studies investigating probiotic interventions against AFB1-induced toxicity via the microbiota–gut–brain axis.

Animal Model	AFB1/Toxins Dose and Route	Probiotic Strain(s)/Formulation	Main Outcomes	Ref.
**SAMP8 mice**	Age-related decline model	ProBiotic-4 (*B. lactis*, *L. casei*, *B. bifidum*, *L. acidophilus*)	Improved memory, reduced neuroinflammation, restored BBB and gut barrier integrity, altered microbiota composition	[106]
**SH-SY5Y cells (in vitro)**	H_2_O_2_-induced oxidative stress	*L. lactis* KC24-CM, *L. rhamnosus* GG, *L. delbrueckii*, *L. plantarum*	Increased BDNF, reduced apoptosis (Bax/Bcl-2), enhanced cell viability	[126]
**N2a cells + C57BL/6 mice (MPP^+^ PD model)**	MPP^+^ neurotoxin	*L. plantarum* CRL2130	Reduced ROS and IL-6, improved motor function, increased IL-10	[129]
**Syrian golden hamsters**	PPA 250 mg/kg ×3 days or clindamycin 30 mg/kg (oral)	Multi-strain mix (*B. infantis*, *B. breve*, *L. acidophilus*, *L. bulgaricus*, *L. casei*, *L. rhamnosus*, *S. thermophilus*)	Restored microbiota balance, improved oxidative stress markers, reduced lipid peroxidation	[103]
**Albino female rats**	AFB1 40 µg/kg feed ×8 weeks	Lactic acid bacteria preparation	Reduced brain lipid accumulation, partial normalization of jejunal and ileal lipid profiles	[131]
**Sprague Dawley rats**	Acrylamide 40 mg/kg/day	*L. plantarum* ATCC8014	↑ SOD, CAT, GSH; ↓ lipid peroxidation; improved histology of brain and gut	[130]
**Adult male rats**	AFB1 25 µg/kg/week ×8 weeks (oral)	VSL#3 (multi-strain, 2.5 × 10^10^ CFU/day)	↑ GSH, GPx, GST, SOD; ↓ MDA, TNF-α, IL-6; fewer anxiety/depression-like symptoms	[94]
**Male mice**	AFB1 51.8 µL/day ×28 days (oral)	*B. amyloliquefaciens* B10	↑ Occludin, claudin-1, ZO-1; ↓ MyD88, TNF-α, IL-6, NF-κB; improved microbiota profile	[132]
***Piaractus mesopotamicus* (fish)**	AFB1 25 or 400 µg/kg feed ×15 days	Probiotic-based adsorbent (PBA)	Improved digestive enzyme activity, reduced gut histopathology	[133]
**C57BL/6J mice (HFD + AFB1)**	AFB1 200 µg/kg/day ×9 weeks (oral)	*B. breve* (10^7^ CFU/day)	↓ Weight gain, improved liver histology, ↑ SCFAs, reduced inflammation, modulated lipid metabolism	[134]

Note: ↑ indicates increased expression or activity; ↓ indicates decreased expression or activity.

**Table 4:** Comparative overview of detection methods for aflatoxins and their metabolites.

Target Analyte	Matrix	Method	LOD	Advantages	Limitations
**AFB1–albumin adducts**	Serum/Plasma	LC–MS/MS after enzymatic digest	pg/mL	High sensitivity/specificity	Requires specialized equipment
**Urinary AFM1**	Urine	ELISA	ng/mL	Fast, high throughput	Less specific vs. chromatographic methods
**Free AFB1/AFM1**	Serum/Milk	HPLC–FLD after SPE cleanup	ng/mL	Established workflow	Time-consuming
**Multiple metabolite profiling**	Plasma/Urine	LC–MS/MS	Low pg/mL	Multiplex capability	High cost, technical requirements
**AFM1 screening**	Urine	LFIA	μg/mL	Portable and rapid	Minimum sensitivity and scope

## Abbreviations

The following abbreviations are used in this manuscript:

AFB1	Aflatoxin B1
AFM1	Aflatoxin M1
AFG1	Aflatoxin G1
AFB2	Aflatoxin B2
AFM2	Aflatoxin M2
AFG2	Aflatoxin G2
EFSA	European Food Safety Authority
FAO	Food and Agriculture Organization of the United Nations
LD_50_	Lethal Dose
CYP450	Cytochrome P450
AFBO	AFB1-exo-8,9-epoxide
WHO	World Health Organization
DT_50_	Dissipation Time
FDA	U.S. Food and Drug Administration
BIS	Bureau of Indian Standards
SOD	Superoxide Dismutase
SGLT1	Sodium-Glucose Cotransporter 1
SLC7A1	Solute Carrier Family 7 Member 1
ZO-1	Zonula Occludens-1
8-OHdG	8-hydroxy-2′-deoxyguanosine
CAT	Catalase
GPx	Glutathione Peroxidase
TAC	Total Antioxidant Capacity
TBARS	Thiobarbituric Acid Reactive Substances
Ca^2+^	Calcium
AST	Aspartate Aminotransferase
ALT	Alanine Aminotransferase
ALB	Albumin
TP	Total Protein
ALP	Alkaline Phosphatase
TNF-α	Tumor Necrosis Factor-alpha
IL-6	Interleukin-6
MDA	Malondialdehyde
T-SOD	Total SOD
GGT	γ-glutamyltransferase
GST	Glutathione S-transferase
NO	Nitric Oxide
GSH	Glutathione
SPF	Specific Pathogen-Free
DMSO	Dimethyl Sulfoxide
b.w.	Body Weight
H_2_O_2_	Hydrogen Peroxide
GR	Glutathione Reductase
I-FABP	Intestinal Fatty Acid-Binding Protein
VH	Villus Height
CD	Crypt Depth
DAO	Diamine Oxidase
PCoA	Principal Coordinate Analysis
VSA	Villus Surface Area
ROS	Reactive Oxygen Species
LPS	Lipopolysaccharide
HCC	Hepatocellular Carcinoma
HBV	Hepatitis B Virus
PAS	Periodic Acid–Schiff
IFN-γ	Interferon-gamma
TLR4	Toll-Like Receptor 4
HIV	Human Immunodeficiency Virus
HDAC	Histone Deacetylase
NOS2	Inducible Nitric Oxide Synthase
APCA	Alternative Pathway of Complement Activation
ACP	Acid Phosphatase
LOAEL	Lowest Observed Adverse Effect Level
BBB	Blood–Brain Barrier
AChE	Acetylcholinesterase
DG	Dentate Gyrus
MAO	Monoamine Oxidase
XO	Xanthine Oxidase
UA	Uric Acid
TH	Tyrosine Hydroxylase
NOR	Novel Object Recognition
GFAP	Glial Fibrillary Acidic Protein
MPO	Myeloperoxidase
RONS	Reactive Oxygen And Nitrogen Species
LPO	Lipid Peroxidation
TSH	Thyroid-Stimulating Hormone
IDO1	Indoleamine 2,3-dioxygenase 1
CNS	Central Nervous System
MAMP	Microbe-Associated Molecular Pattern
SCFA	Short-Chain Fatty Acid
ADHD	Attention-Deficit Hyperactivity Disorder
BDNF	Brain-Derived Neurotrophic Factor
SAMP8	Senescence-Accelerated Prone 8
EPS	Exopolysaccharides
Aβ	Amyloid Beta
MPP^+^	1-methyl-4-phenylpyridinium
PPA	Propionic Acid
LDH	Lactate Dehydrogenase
AA	Acrylamide
ELISA	Enzyme-Linked Immunosorbent Assay
LFIA	Lateral Flow Immunoassay
HPLC-FLD	High-Performance Liquid Chromatography with Fluorescence Detection
UHPLC	Ultra-High-Performance Liquid Chromatography
LC-MS/MS	Liquid Chromatography–Tandem Mass Spectrometry
SPE	Solid-Phase Extraction
QuEChERS	Quick, Easy, Cheap, Effective, Rugged, and Safe
